# Single Center, Propensity Score Matching Analysis of Different Reconstruction Techniques following Pancreatoduodenectomy

**DOI:** 10.3390/jcm12093318

**Published:** 2023-05-06

**Authors:** Ruben Bellotti, Benno Cardini, Carola J. Strolz, Stefan Stättner, Rupert Oberhuber, Eva Braunwarth, Thomas Resch, Stefan Scheidl, Christian Margreiter, Stefan Schneeberger, Dietmar Öfner, Manuel Maglione

**Affiliations:** 1 Department of Visceral, Transplant and Thoracic Surgery, Center of Operative Medicine, Medical University of Innsbruck, 6020 Innsbruck, Austria; ruben.bellotti@i-med.ac.at (R.B.);; 2 Department of General, Visceral and Vascular Surgery, Salzkammergut Hospital, 4840 Vöcklabruck, Austria

**Keywords:** pancreatogastrostomy, pancreatojejunostomy, propensity score, pancreatic fistula, fistula risk score, post-pancreatectomy hemorrhage

## Abstract

Background: Pancreatoduodenectomy is still hampered by significant morbidity. So far, there is no universally accepted technique aimed at minimizing postoperative complications. Herein, we compare three different reconstruction techniques. Methods: This is a retrospective study of a prospectively maintained database including 283 patients operated between January 2010 and December 2020. Three reconstruction techniques were compared: (1) the Neuhaus-style telescope pancreatojejunostomy, (2) the pancreatogastrostomy, and (3) the modified Blumgart-style, duct-to-mucosa pancreatojejunostomy. The primary endpoint consisted in determining the rates of clinically relevant postoperative pancreatic fistulas (CR-POPF); the secondary endpoints included 90 days morbidity and mortality rates. A propensity score matching analysis was used. Results: Rates of CR-POPF did not differ significantly between the groups (Neuhaus-style pancreatojejunostomy 16%, pancreatogastrostomy 17%, modified Blumgart-style pancreatojejunostomy 15%), neither in the unmatched nor in the matched analysis (*p* = 0.993 and *p* = 0.901, respectively). Similarly, no significant differences could be observed with regard to major morbidity (unmatched *p* = 0.596, matched *p* = 0.188) and mortality rates (unmatched *p* = 0.371, matched *p* = 0.209) within the first 90 days following surgery. Propensity-score matching analyses revealed, however, a higher occurrence of post-pancreatectomy hemorrhage after pancreatogastrostomy (*p* = 0.015). Conclusion: Similar CR-POPF rates suggest no crucial role of the applied reconstruction technique. Increased incidence of intraluminal post-pancreatectomy hemorrhages following pancreatogastrostomy demands awareness for meticulous hemostasis.

## 1. Introduction

Pancreatoduodenectomy (PD) is still hampered by significant morbidity varying between 20% and 50% [1,2]. The most feared complication is represented hereby by the clinically relevant postoperative pancreatic fistula (CR-POPF). The occurrence of CR-POPF is associated with erosion bleedings, sepsis, and multiple organ failure, necessitating further invasive treatments [3], and even resulting in patient death in up to 5% [4,5].

Aimed at minimizing the occurrence of CR-POPF, numerous techniques to restore the continuity between the pancreatic stump and the gastro-enteric tract following PD have been proposed. However, so far, no universally accepted reconstruction procedure has shown a clear superiority. Recent prospective randomized controlled trials (RCTs) as well as retrospective analyses comparing different reconstruction techniques resulted in discordant conclusions and lacked in identifying a technique able to clearly decrease the occurrence of CR-POPF [3,6,7,8,9,10,11,12,13,14,15,16,17]. However, both performed RCTs emphasized the higher incidence of major complications following pancreatogastrostomy (PG), with Andrianello et al., showing that PG was associated with an increased burden of complications in patients developing CR-POPF and suggesting PJ as an appropriate reconstruction technique for patients at high risk of POPF [6]. 

Not only the use of different anastomosis techniques but also the application of several mitigation strategies has been suggested in the past years to reduce the rates of CR-POPF. While the efficacy of sealants or of an omental roll-up in preventing postoperative morbidity is still a matter of debate [18,19,20], the use of external drainage of the pancreatic duct showed improved rates of CR-POPF [21,22,23]. Moreover, contrasting results concerning POPF have been achieved using somatostatin analogs [6,24,25]. 

Over the last ten years, three different reconstruction techniques following PD have been performed at our department. Rather than deciding on the basis of specific organ features such as the consistency of the pancreatic parenchyma or the size of the main pancreatic duct, the different techniques represent three different historical phases of the department. Two dunking or telescope techniques and one duct-to-mucosa technique were used. First, the one-layered telescope pancreatojejunostomy using inverted mattress sutures according to Neuhaus [26] was performed, then a one-layered pancreatogastrostomy [27], and in the third period, the modified duct-to-mucosa, Blumgart-style pancreatojejunostomy completed with transpancreatic sutures to cover the cut pancreatic surface with jejunal serosa [28]. 

This study aimed to review postoperative outcomes focusing on the three different reconstruction techniques performed at our institution with regard to the rate of CR-POPF as well as the overall complication rate. To obviate shortcomings of the retrospective character, a propensity score matching model, based on an inverse probability of treatment weights (IPTWs) [29] was applied. 

## 2. Materials and Methods

A retrospective analysis of prospectively collected and auditable medical records from all patients who underwent a PD between January 2010 and December 2020 at the Department of Visceral, Transplant and Thoracic Surgery, Innsbruck, Austria, was performed. Reporting is consistent with the STROBE guidelines [30] for observational research and the principles of the Declaration of Helsinki. 

### 2.1. Data Collection and Cohort Selection 

Data collected included baseline demographics and clinical characteristics, fistula risk score (FRS) [31], intraoperative aspects such as portal vein/superior mesenteric vein resection, CR-POPF as defined by the International Study Group on Pancreatic Surgery (ISGPS) [32], major complications (defined as Clavien–Dindo classification ≥ 3a) [33], hospital readmission, and mortality rates, all within 90 days following surgery.

**Figure 1:**
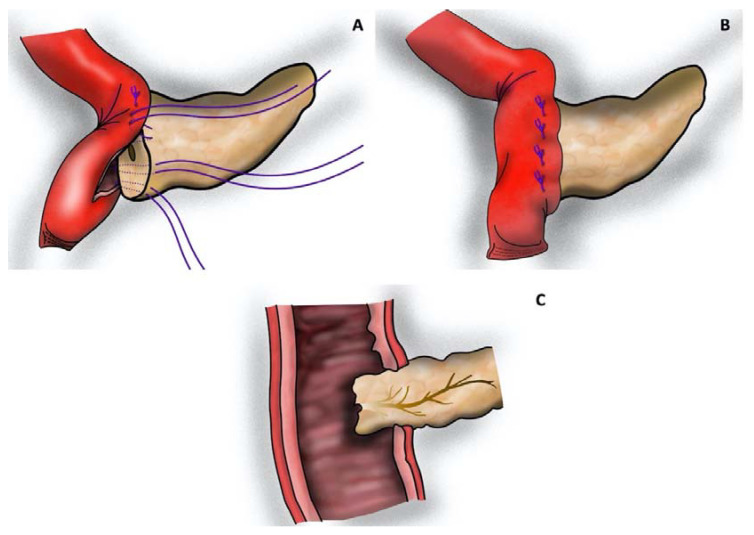
Neuhaus-style telescope pancreatojejunostomy (tsPJN): (**A**) U-shaped sutures running through the jejunal wall from back to front straight through the pancreatic parenchyma; (**B**) tied U-shaped sutures placed through the front wall of the jejunal loop; and (**C**) transverse section. The pancreas stump becomes completely enclosed by the jejunal loop. (Picture realized with Affinity Designer 1.10.5).

**Figure 2:**
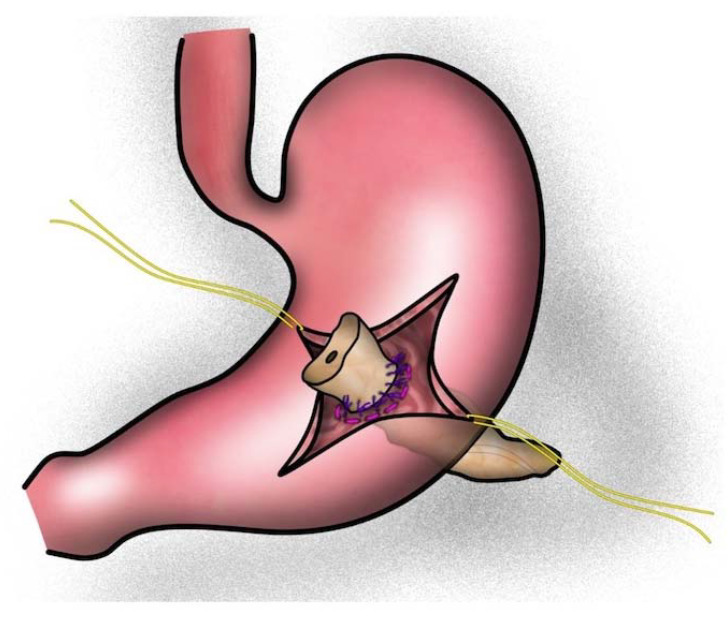
Pancreatogastrostomy (PG): The anastomosis is carried out under direct vision following an anterior gastrostomy. The pancreatic stump is brought through a posterior gastric incision and sutured to the posterior pancreatic wall. (Picture realized with Affinity Designer 1.10.5).

**Figure 3:**
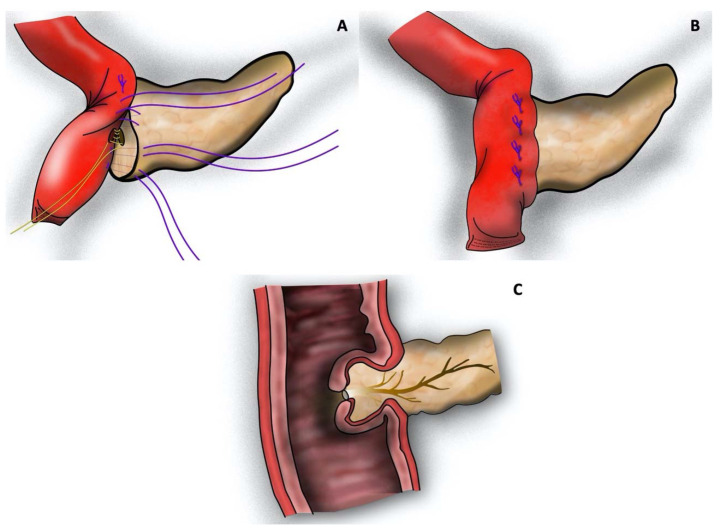
Modified Blumgart-style, duct-to-mucosa pancreatojejunostomy (dtmPJB): (**A**) U-shaped sutures running through the pancreatic parenchyma from the anterior to the posterior side, through the seromuscular layer of the jejunal loop, and then back from the posterior to the anterior side of the pancreatic remnant. The duct-to-mucosa anastomosis is sutured with 6 interrupted sutures; (**B**) tied U-shaped sutures brought through the seromuscular layer of the jejunal loop; and (**C**) transverse section. The cut pancreatic surface is covered by the jejunal loop. (Picture realized with Affinity Designer 1.10.5).

Patients were divided into three groups according to the reconstruction technique used: (1) one-layered telescope pancreatojejunostomy using inverted mattress sutures according to Neuhaus (tsPJN, Figure 1) [26], (2) one-layered pancreatogastrostomy (PG, Figure 2) [27], or (3) modified Blumgart-style, duct-to-mucosa pancreatojejunostomy (dtmPJB, Figure 3) [28].

The primary endpoint of the study was defined as the rate of CR-POPF. Secondary endpoints were the incidence of delayed gastric emptying (DGE), post-pancreatectomy hemorrhage (PPH), and biliary fistula, as well as the rates of major complications (Clavien–Dindo ≥ 3a) and mortality within the first 90 days after surgery [26].

### 2.2. Surgical Procedures

All PDs were performed by experienced surgeons. Excluding the years 2011, 2014, and 2015, more than 20 PDs per year were regularly performed at our institution with up to three surgeons operating the yearly occurring caseload. They were mostly performed according to the Traverso–Longmire procedure [34], and only in 11 cases, due to suspected tumoral invasion of the pyloric region, the Whipple–Kausch technique was used [35]. Standard lymphadenectomy was regularly performed according to the guidelines of the International Study Group on Pancreatic Surgery (ISGPS) [36]. In the Supplementary Materials (Figure S1), time distribution and caseload for each anastomosis group are depicted.

The tsPJN was performed as originally described [26]. In brief, an antimesenteric incision of the jejunal loop is performed, similar in size to the resection surface of the pancreatic stump. Starting at the back wall of the jejunum, U-stitches are placed through the jejunal wall and from back to front straight through the pancreatic parenchyma, 1 cm distally from the cut surface. Finally, the sutures are placed through the front wall of the jejunal loop. In order not to occlude the pancreatic duct, an externally draining stent is inserted. Finally, resembling the ‘‘telescope’’ or ‘‘dunking’’ technique [37], the pancreas stump becomes completely enclosed by the jejunal loop (Figure 1).

Concerning the pancreatic drain, the external end is guided through the intestinal wall 20 cm distally from the pancreatic anastomosis using a small metal trocar. Following fixation using the Witzel tunnel technique, the external end is finally guided through the abdominal wall with the metal trocar before abdominal closure.

The PG was performed as previously published [38]. Following 4 cm mobilization of the pancreatic remnant, a horizontal incision is performed on the posterior gastric wall and a purse-string suture is placed. After that, the anastomosis can be carried out under direct vision following an anterior gastrostomy. The pancreatic stump is brought through the posterior gastric incision and sutured to the posterior gastric wall. An internal stent is applied to avoid occlusion of the pancreatic duct. Finally, the anterior gastrostomy is closed with a two-layer continuous suture (Figure 2).

The dtmPJB was slightly modified compared to the original publication [28]. Briefly, using 4 U-sutures, the needle is passed through the pancreatic stump from the anterior to the posterior side, and then, in a parallel fashion, through the seromuscular layer of the jejunal loop, and finally back from the posterior to the anterior side of the pancreatic remnant. Then, a small jejunostomy is performed and the duct-to-mucosa anastomosis is sutured using 6 interrupted sutures. An externally draining pancreatic stent is inserted as described above. 

The last step of the procedure consists in passing the U-sutures through the seromuscular layer of the jejunum before tying them. Hereby, the cut pancreatic surface is entirely covered by the jejunal loop (Figure 3). In the original procedure, sutures were tied also on the anterior face of the pancreatic parenchyma before passing them through the seromuscular layer of the jejunal loop.

Mitigation strategies such as perioperative somatostatin or positioning of an omental roll-up around the pancreatic anastomosis were performed depending on the surgeons’ preferences. In all patients, prophylactic antibiotics and low molecular weight heparin were administered. The amylase and lipase levels in drain fluid were measured daily. Levels of these enzymes were considered pathological if more than threefold higher than the serum levels on or after postoperative day 3 [32]. In the case of normal amylase and lipase levels in the drainage and drainage output <50 mL in the last 24 h, this was removed on the following day. 

The externally draining pancreatic stents (in tsPJN and dtmPJB) were removed 6 weeks following PD.

### 2.3. Statistical Analysis

Statistical analysis was performed using R 2016 statistical software (Team RC, Foundation for Statistical Computing, Vienna, Austria) [39]. Continuous variables are reported as median (range) and categorical variables as frequency and percentage. A univariate analysis to compare the three different anastomosis techniques was conducted. The Pearson’s chi-squared test and Wilcoxon test were used in the univariate analysis. Regression analyses, more specifically, likelihood ratio chi-square tests (LR-Chisq), were used to perform the comparative analysis of clinical outcomes between the three anastomosis groups. Two-tailed *p*-values less than 0.05 were considered statistically significant. 

To overcome biases due to observed confounders among preoperative variables, we performed a matched propensity score (PS) analysis to assess treatment effects. A logistic regression model including all the covariates from Table 1 was used to estimate the PS. 

Following recommendations in the current literature [40,41], weighting was performed using IPTW [42]. To control all possible confounders in our regression model, we adjusted the analysis for all of them using the application of a weighting factor calculated with the PS. A treated patient with a low PS (for the treatment) receives a high weighting because he/she is similar to an untreated patient in terms of his/her characteristics (expressed as his/her low PS), so a valid comparison can be made between the two. For the evaluation of the treatment effect, patients enter the statistical analysis according to their weight. 

Case matching with IPTW allows the inclusion of all study subjects in the matched analysis and is applied to estimate the Average Treatment Effect (ATE), hence, overcoming the bias of a small observational study with many confounders.

Balance measurements were conducted for each group against the other two groups. The overall assessment of the discrimination was evaluated using the z-difference, with larger values indicating a better discrimination [29]. Propensity scoring and matching were conducted using the twang package version 2.5 for R software version 4.1.2. The estimation method was performed with ATE, and the stop method was the effect size (ES) and the Kolmogorov–Smirnov (KS) statistic mean [43].

## 3. Results

This study included 282 patients undergoing PD. In total, 116 patients underwent a tsPJN, 75 a PG, and 91 a dtmPJB. One patient was excluded from the general statistical analysis because of an incomplete data set. 

Regarding the demographic data, the unmatched analysis revealed some statistically significant differences between the three groups such as pre-existing diabetes, alcohol consumption, neoadjuvant chemotherapy, ASA classification, and preoperative biliary drainage (ERCP and/or PTCD) as well as endoscopic stenting. However, following the PS analysis with IPTW estimation, there were no more statistically significant differences between the analyzed groups. 

Similarly, mitigation strategies did not differ significantly between the three reconstruction techniques. With regard to the different items in the FRS, only the intraoperative blood loss showed a statistical significance with a higher number of patients losing more than 400 mL of blood in the PG group. The low number of patients with portal/mesenteric reconstruction and those where sealants were applied did not allow any further analysis (Table 1).

Chronic infections including any stable bacterial/fungal colonization of the skin (decubitus) or the mucosa (candidiasis) and jaundice and cholangitis were excluded. Previous malignancies included any liquid or solid tumor in the past patient history (before pancreatic surgery). Low-risk pathologies according to the FRS were pancreatic ductal adenocarcinoma and chronic pancreatitis. High-risk pathologies according to the FRS were ampullary cancer, duodenal carcinoma, cholangiocarcinoma of the distal bile duct, neuroendocrine neoplasms, any cystic lesions, benign entities, and distant metastases to the pancreas (specific diagnosis are listed in the Supplementary Table S1).

The postoperative complication rates are indicated in Table 2. Regarding the primary study endpoint, tsPJN, PG, and dtmPJB resulted in a similar rate of CR-POPF (16% vs. 17% vs. 15%, respectively) and showed no significant differences in the unmatched analysis (*p* = 0.993). The PS confirmed the missing significant differences in CR-POPF rates between the three groups (*p* = 0.901). 

Similarly, no significant differences could be detected for the occurrence of biliary fistulas (unmatched *p* = 0.607; matched *p* = 0.253) or DGE (unmatched *p* = 0.682; matched *p* = 0.441). Of note, PG showed numerically higher DGE rates (21% PG vs. 16% tsPJN vs. 14% dtmPJB, respectively).

While there was no statistical significance in the unweighted analysis (*p* = 0.556), PS matching revealed, that PG was associated with significantly higher PPH of any grade compared to both PJs (*p* = 0.015). The difference was still there when comparing each single PPH grade between the three different techniques; however, the higher occurrence in the PG group did not reach statistical significance (*p* = 0.064).

Neither of the three techniques revealed a significant reduction in severe complications (Clavien–Dindo ≥ 3a) both in the unmatched and matched analyses (*p* = 0.596 and *p* = 0.188, respectively). Equally, both analyses showed no difference concerning 90 days mortality between the three different techniques (*p* = 0.371 and *p* = 0.209, respectively), even if restricted to cases with POPF type C (*p* = 0.682 and *p* = 0.209, respectively).

The median follow-up time for our patients was 30 months (range 0–134) and differed significantly between the three study groups (tsPJN 33 months; PG 45 months; dtmPJB: 18 months, respectively; *p* < 0.001).

## 4. Discussion

To our knowledge, this is the first retrospective study comparing one PG with two different PJ techniques following PD using an IPTW-based PS matching. The general occurrence of CR-POPF is in line with that of other reporting centers [3,16,17]. Using the IPTW-based PS matching, we could not observe any differences between the analyzed reconstruction techniques concerning the rate of CR-POPF. None of the three analyzed pancreatic anastomosis techniques resulted in a significant advantage, neither concerning the occurrence of CR-POPF in general nor specifically concerning grade C fistulas. 

Our findings reflect the currently available literature [44], where no specific reconstruction technique after PD was demonstrated to be clearly superior concerning the rates of CR-POPF. Publications are, in fact, conflicting. While the comparison between PJ and PG did not show any significant difference in a recent randomized controlled trial (RCT) [3,6] and in recent meta-analyses [9,14,15], previous publications [45,46] showed the superiority of PG compared to PJ in preventing this complication. 

On the same line, studies focusing solely on different PJ techniques also present heterogeneous results. A recent retrospective study including 110 patients and using PS matching suggested a minimization of grade C POPF using the Blumgart technique [16]. These findings are supported by some meta-analyses and other retrospective studies showing that the Blumgart-style anastomosis is more effective in preventing CR-POPF compared to other PJ invagination techniques [7,8,12,17], even for high-risk subjects [13]. In contrast, no significant differences in preventing CR-POPF could be observed in an RCT [13], a meta-analysis [14], and two other retrospective studies [10,11]. These contrasting results presumably reflect various biases such as lack of statistical power, the mostly retrospective, single-center nature of the studies, and, last but not least, also the different modifications of the described anastomosis techniques, which makes a direct comparison impossible.

The presented data are also consistent with those from the RECOPANC study [3], which is one of the largest RCTs conducted on this topic. Not restricted to a specific PJ or PG technique, in this RCT, both reconstruction strategies, PJ as well as PG, shared analogous CR-POPF rates. 

The risk to develop a CR-POPF is classically calculated using different FRS. Parenchymal texture, dimensions of the main pancreatic duct at the cut surface, intraoperative blood loss, underlying pathology, neoadjuvant treatment, and also minimal-invasive techniques are all recognized factors influencing the occurrence of POPF and were considered in the different FRS developed over the last years [31,47,48]. Unfortunately, the parenchymal structure is poorly documented in our database. Therefore, the exact FRS could only be calculated in less than half of the included patients (135/283). Due to the questionable validity of a sub-analysis on a small patient cohort, we decided to limit the FRS analysis by defining the number of patients presenting a high FRS in each group. With only the parenchymal structure missing in 148 patients, an indirect calculation able to identify FRS 7–10 was possible, resulting finally in the characterization of almost all (278/283) patients.

In our cohort, only very few patients showed a high classical FRS [31], most of them occurring in the PG group. With all items except intraoperative blood loss being different between the groups, the higher number of patients presenting high FRS in the PG group depended mostly on this item rather than on pancreas characteristics.

The use of such scores and their clinical relevance was the object of recent debates. In an analysis of different externally validated fistula scores and their comparison with each other, the PARANOIA study group showed that these are only of limited advice regarding their predictive accuracy [49]. Along the same line, a newly FRS comprehensive catalog showed that rather than a single numeric score, an improved granularity resulting in 80 different FRS scenarios would outperform the existing FRS. Of note, the 10 most clinically impactful scenarios for POPF development included FRS ranging from 2 to 7; however, all of them included a soft gland. Interestingly, this study group showed the superiority of PJ in the upper values of the moderate risk zone of the FRS [50].

With regard to the effect of neoadjuvant radiotherapy and the use of mitigation strategies such as the use of sealants, omental roll-up, and perioperative somatostatin, treatment sample size did not allow any further analysis for the impact of these factors on CR-POPF occurrence. All patients received either an internal (PG) or an externally draining (tsPJN and dtmPJB) pancreatic stent. This resulted in similar rates of CR-POPF for all techniques, supporting data showing no difference between the two stenting strategies [51].

Even regarding the occurrence of biliary fistulas, there was not any significant difference in the three analyzed patient groups. This is in line with other studies that did not find any relation between the pancreatic anastomosis technique and early biliary complications more in general [52] or between different reconstruction techniques and biliary fistula/leakage [53]. 

Reported rates of DGE following PD range from 5 to 59% [54]. Even though PG is mostly considered to be a risk factor for DGE, findings regarding its association with a certain type of pancreatic remnant reconstruction are conflicting [55,56,57]. In our cohort, DGE rates were comparable between the three groups, both in the unmatched and matched analyses. Nevertheless, the PG group showed a considerably higher trend toward DGE development.

Another particularity observed in the PS analysis was the higher occurrence of any grade of PPH following PG reconstruction. Of note, despite the comparable rates of PV/SMV resection and therapeutic anticoagulation in all groups, the intraoperative bleeding rates of the PG group were significantly higher. These results are concordant with the RECOPANC study [3] and also with the recent RCT considering patients with high risk for CR-POPF [6]. Considering the PPH classification of the ISGPS [58], the wide majority of bleedings after PG in our series occurred intraluminal within the stomach (17 out of 23). Conversely, patients becoming any type of PJ showed bleeding episodes mostly in correspondence with the gastroduodenal artery, the pancreatic arterial arcades, and the hepatic artery. This finding could be related to the conspicuous vascularization of the gastric wall. 

Concerning major surgical morbidity rates, a recent PS matching analysis comparing three different PJs showed that the Blumgart-anastomosis could reduce them [16]. Similar results have been also reported by other studies [8,17]. The superiority of the Blumgart technique is thought to be related to the reduction in tangential tension and shear force at the pancreatic stump. This should guarantee better blood supply to the remnant if compared to other types of pancreatojejunostomies. However, the originally described technique has repeatedly been subject to critical debate. Kim et al., argued that the originally reported Blumgart technique did not completely cover the pancreatic remnant with the jejunal loop, therefore bearing an even increased risk for CR-POPF [59]. Therefore, a plethora of variants of this technique have been proposed, and the resulting inhomogeneity leads to a difficult standardization and comparison of the reported data regarding surgical morbidity.

On the contrary, the RECOPANC study showed no significant correlation between any type of pancreatic anastomosis and surgical complications other than PPH [3]. Similarly, in the present study, the occurrence of severe complications (Clavien–Dindo ≥ 3a) was not related to the anastomosis technique. 

Several studies highlight the importance of center volume when discussing mortality and major morbidities as well as the failure to rescue rates following pancreatic resections. A nationwide comparison between German and Dutch audits highlights a significant center volume–outcome relationship concerning in-hospital mortality for very low- and low-volume centers (defined as ≤20 procedures per year) [60]. Similarly, two recent nationwide analyses on major pancreatic surgery in Germany and Italy suggest a strong relationship between volume center and in-hospital mortality rates, advocating new centralization policies based on volume and mortality thresholds [61,62]. The results presented in the present study go along with this point of view, where the focus should be more on numbers and standardization rather than on the reconstruction techniques itself. In fact, during the reported time, most surgeons performed not only one but at least two of the three reconstruction techniques without observing major differences in the rates of postoperative complications. Our data suggest that surgical experience combined with an adequate caseload seems to be more important than choosing a specific reconstruction technique. However, the development of new techniques should not be neglected when aiming at improving patient outcomes. In the last years, new techniques applying purse-string sutures on the jejunal opening [63] or around the pancreatic duct [64] have been described as showing promising results considering the rates of CR-POPF. Since the published caseload available is still limited, further studies are needed to validate the superiority of these techniques.

There are several limitations of this study, including incomplete documentation of the pancreatic texture as well as the relatively small number of patients per year over a relatively long “recruiting” time. The statistically significant difference in follow-up times among the different study groups reflects the different time periods when different anastomosis techniques were performed. Regarding postoperative records within the first 90 days, we could only define the presence of postoperative diabetes (type IIIc) according to the need for anti-diabetic drugs and by observing the mean fasting glycemia of the whole cohort. Unfortunately, records concerning HbA1c could only be retrieved for 50 patients. This sub-cohort of patients with no diabetes at the time of diagnosis and developing type IIIc diabetes after PD showed no differences in relation to the anastomosis technique (32.3% for tsPJN, 20.3% for PG, and 27.8% for dtmPJB, respectively, and *p* = 0.239). Still, the low number of patients presenting with postoperative diabetes precludes any apodictic conclusions.

Regarding the inherent limitations of retrospective databases, we tried to compensate for this bias using the IPTW-based PS analysis, which is a useful tool to diminish possible confounder-related errors, and which also allows the inclusion of all analyzed patients. PS matching has been increasingly used in the surgical literature in the last decade to balance patient groups across known risk factors and confounders, representing a reasonable tool to overcome the shortcomings of retrospective analyses [65]. 

## 5. Conclusions

Our study compares three different pancreatic anastomosis techniques (one PG and different PJs) using techniques using an IPTW-based, PS-matched analysis. The presented data suggest that the applied reconstruction technique following pancreatoduodenectomy does not necessarily represent a crucial factor with regard to CR-POPF occurrence. These results are in line with those of a recent RCT and thus confirm the validity and reliability of PS-matched analysis on retrospective databases. The increased incidence of intraluminal postoperative bleedings following PG in these patients demands special awareness concerning intraoperative hemostasis but also during the early postoperative period.

## Figures and Tables

**Table 1:** Demographic, Clinical/Pretreatment, Operative, and Organ Characteristics.

Factors		Type of Anastomosis			Unmatched		Matched	
	All (*n* = 282)	tsPJN (*n* = 116)	PG (*n* = 75)	dtmPJB (*n* = 91)	z	*p* ^†^	z	*p* ^†^
Age *	66 (56–73)	65 (54–71)	66 (56–73)	68 (60–76)	0.42	0.003	0.16	0.254
Sex Ratio (M:F)	164:118	63:53	43:32	58:33	0.19	0.174	0.10	0.489
BMI (kg/m^2^) *	24.4 (22.2–27.3)	24.4 (21.8–27.4)	24.6 (22.9–27.6)	24.4 (22.1–26.9)	0.13	0.328 ^§^	0.04	0.772
ASA (1–5)					0.61	0.000	0.29	0.084
1–2	180 (64.0)	76 (65.5)	59 (79.0)	45 (49.0)				
3–4	102 (36.0)	40 (34.5)	16 (21.0)	46 (51.0)				
Tobacco Smoke	93 (33.0)	46 (39.7)	22 (29.3)	25 (27.5)	0.26	0.069	0.18	0.255
Alcohol Consumption					0.37	0.003	0.24	0.134
Occasionally	75 (26.6)	29 (25.0)	28 (37.3)	18 (19.8)				
Abuse	22 (7.8)	18 (15.5)	1 (1.3)	3 (3.3)				
Pulmonary Disease	46 (16.0)	20 (17.2)	12 (16.0)	14 (15.4)	0.05	0.721	0.14	0.887
Diabetes	49 (17.4)	24 (21.0)	6 (8.0)	19 (21.0)	0.34	0.020	0.25	0.124
Cardiovascular Disease	157 (55.7)	70 (60.0)	43 (57.0)	44 (48.0)	0.24	0.087	0.19	0.228
Chronic Metabolic Disease (not Diabetes)	105 (37.0)	50 (43.1)	24 (32.0)	31 (34.1)	0.23	0.127	0.09	0.532
Previous Malignancies	48 (17.0)	18 (16.0)	12 (16.0)	18 (20.0)	0.11	0.424	0.04	0.785
Chronic Infections	10 (4.0)	6 (5.2)	2 (2.7)	2 (2.2)	0.16	0.273	0.14	0.368
Neoadjuvant Chemotherapy	14 (5.0)	3 (3.0)	2 (3.0)	9 (10.0)	0.34	0.027	0.21	0.204
Neoadjuvant Radiotherapy	1 (0.4)	0 (0.0)	0 (0.0)	1 (1.1)	n.a.	n.a.	n.a.	n.a.
Serum Bilirubin (mg/dl) *	0.83 (0.42–3.52)	0.77 (0.43–2.38)	0.96 (0.49–6.36)	0.74 (0.40–4.55)	0.24	0.129 ^§^	0.72	0.466
Biliary Drainage								
ERCP	106 (37.6)	56 (48.0)	20 (27.0)	30 (33.0)	0.45	0.003	0.21	0.204
PTCD	10 (8.6)	1 (1.3)	3 (3.3)	10 (8.6)	0.34	0.037	0.22	0.138
With stenting ^$^	79 (28.0)	37 (32.0)	14 (19.0)	28 (31.0)	0.29	0.046	0.20	0.241
Endoscopic Biopsy	103 (36.5)	58 (50.0)	21 (28.0)	24 (26.0)	0.49	0.001	0.27	0.070
PV/SMV Resection	42 (14.9)	14 (12.1)	9 (12.0)	19 (20.9)	0.24	0.088	0.16	0.277
PV Resection ^‡^					n.a.	n.a.	n.a.	n.a.
Wedge excision	9 (3.2)	1 (0.9)	4 (5.3)	4 (4.4)				
End to end reconstruction	19 (6.7)	7 (6.0)	4 (5.3)	8 (8.8)				
Prothesis	2 (0.7)	1 (0.9)	0 (0.0)	1 (1.0)				
SMV Resection ^‡^					n.a.	n.a.	n.a.	n.a.
Wedge excision	6 (2.1)	3 (2.6)	1 (1.3)	2 (2.2)				
End to end reconstruction	5 (1.8)	1 (0.9)	0 (0.0)	4 (4.4)				
Prothesis	1 (0.4)	1 (0.9)	0 (0.0)	0(0.0)				
Use of Sealants	78 (27.7)	52 (44.8)	19 (25.3)	7 (7.7)	0.829	0.001	0.30	0.070
Omental roll-up	37 (13.1)	13 (11.2)	11 (14.7)	13 (14.3)	0.245	0.744	0.09	0.573
Therapeutic anticoagulation	52 (18.4)	17 (14.7)	13 (17.3)	22 (24.2)	0.102	0.999	0.09	0.575
Somatostatin perioperative ^‡^	10 (3.5)	1 (0.9)	1 (1.3)	8 (8.8)	0.428	0.906	n.a.	n.a.
Parenchyma Texture (*n* = 135)					0.71	0.476	1.53	0.126
Soft	67 (49.6)	24 (53.3)	10 (34.5)	33 (54.1)				
Hard	68 (50.4)	21 (46.7)	19 (65.5)	28 (45.9)				
High-risk Pathology ^#^	125 (44·0)	49 (42·2)	38 (50·7)	38 (41·8)	0.18	0.255	0.13	0.438
Duct Size					0.08	0.557	0.07	0.661
>5 mm	0 (0.0)	0 (0.0)	0 (0.0)	0 (0.0)				
4 mm	168 (59.6)	71 (61.2)	45 (60.0)	52 (57.1)				
3 mm	13 (4.6)	4 (3.4)	3 (4.0)	6 (6.6)				
2 mm	97 (34.4)	39 (33.6)	25 (33.3)	33 (36.3)				
<1 mm	4 (1.4)	2 (1.7)	2 (2.7)	0 (0.0)				
Intraoperative Blood Loss					0.43	0.011	0.20	0.024
<400 mL	218 (77.3)	91 (78.4)	54 (72.0)	73 (80.2)				
400–700 mL	61 (21.6)	24 (20.7)	20 (26.7)	17 (18.7)				
700–1000 mL	2 (0.7)	1 (0.9)	1 (1.3)	0 (0.0)				
>1000 mL	1 (0.4)	0 (0.0)	0 (0.0)	1 (1.1)				
FRS (*n* = 278) ^‡^					n.a.	n.a.	n.a.	n.a.
0–6	257 (92.4)	108 (93.9)	64 (85.3)	85 (96.6)				
7–10	21 (7.6)	7 (6.1)	11 (14.7)	3 (3.4)				

Values in parenthesis are percentages unless indicated otherwise; * values are median (range). ^†^ χ^2^ test except; ^§^ Wilcoxon test; ^$^ Includes both ERCP and/or PTCD stenting; ^#^ According to the fistula risk score (FRS); ^‡^ analysis not applicable due to either too small sample size (somatostatin) or due to direct dependence from other included variables (PV, SMV, and FRS). tsPJN: Neuhaus-style telescope pancreatojejunostomy; PG: pancreatogastrostomy; dtmPJB: modified Blumgart-style, duct-to-mucosa pancreatojejunostomy; BMI: body mass index; ASA: American Society of Anesthesiologists classification; ERCP: endoscopic retrograde cholangiopancreatography; PTCD: Percutaneous transhepatic cholangiography; PV: portal vein; SMV: superior mesenteric vein; FRS: fistula risk score. Chronic Metabolic Disease: any condition altering the normal metabolism such as thyroid dysfunction or dyslipidemia.

**Table 2:** Postoperative Complications.

	All (*n* = 282)	tsPJN (*n* = 116)	PG (*n* = 75)	dtmPJB (*n* = 91)	Unw. ^†^	Weights ^‡^
POPF					χ^2^_(2)_ = 0.12, *p* = 0.941	χ^2^_(2)_ = 0.19, *p* = 0.910
0 (zero)	223 (79.0)	91 (78.4)	60 (80.0)	72 (79.1)		
A	13 (5.0)	6 (5.2)	2 (2.7)	5 (5.5)		
B	27 (10.0)	13 (11.2)	5 (6.6)	9 (9.9)		
C	19 (7.0)	6 (5.2)	8 (10.7)	5 (5.5)		
POPF (grouped)					χ^2^_(2)_ = 0.11, *p* = 0.993	χ^2^_(2)_ = 0.21, *p* = 0.901
0 (zero)/A	236 (83.7)	97 (83.6)	62 (82.7)	77 (84.6)		
B/C	46 (16.3)	19 (16.4)	13 (17.3)	14 (15.4)		
Biliary Fistula					χ^2^_(2)_ = 1.00, *p* = 0.607	χ^2^_(2)_ = 2.75, *p* = 0.253
0 (zero)	269 (95.4)	112 (96.6)	73 (97.3)	84 (92.3)		
Conservative Management	1 (0.4)	1 (0.9)	0 (0.0)	0 (0.0)		
Drainage	1 (0.4)	0 (0.0)	0 (0.0)	1 (1.1)		
Reoperation	11 (3.9)	3 (2.6)	2 (2.7)	6 (6.6)		
DGE					χ^2^_(2)_ = 0.77, *p* = 0.682	χ^2^_(2)_ = 1.64, *p* = 0.441
0 (zero)	235 (83.0)	98 (84.5)	59 (78.7)	78 (85.7)		
A	23 (8.0)	6 (5.2)	8 (10.7)	9 (9.9)		
B	14 (5.0)	7 (6.0)	5 (6.7)	2 (2.2)		
C	10 (4.0)	5 (4.3)	3 (4.0)	2 (2.2)		
DGE (grouped)					χ^2^_(2)_ = 0.20, *p* = 0.907	χ^2^_(2)_ = 1.31, *p* = 0.520
0 (zero)	235 (83.3)	98 (84.5)	59 (78.7)	78 (85.7)		
A + B + C	47 (16.7)	18 (15.5)	16 (21.3)	13 (14.3)		
PPH					χ^2^_(2)_ = 4.16, *p* = 0.125	χ^2^_(2)_ = 5.49, *p* = 0.064
0 (zero)	225 (80.0)	93 (80.2)	52 (69.3)	80 (87.9)		
A	10 (4.0)	4 (3.4)	5 (6.7)	1 (1.1)		
B	23 (8.0)	11 (9.5)	8 (10.7)	4 (4.4)		
C	24 (9.0)	8 (6.9)	10 (13.3)	6 (6.6)		
PPH (grouped)					χ^2^_(2)_ = 1.18, *p* = 0.556	χ^2^_(2)_ = 8.37, *p* = 0.015
0 (zero)	225 (79.8)	93 (80.2)	52 (69.3)	80 (87.9)		
A + B + C	57 (20.2)	23 (19.8)	23 (30.7)	11 (12.1)		
Wound Complication	39 (14.0)	16 (13.8)	14 (18.7)	9 (9.9)	χ^2^_(2)_ = 2.65, *p* = 0.266	χ^2^_(2)_ = 3.20, *p* = 0.202
90 days Relaparotomy	49 (17.4)	16 (13.8)	15 (20.0)	18 (19.8)	χ^2^_(2)_ = 1.80, *p* = 0.406	χ^2^_(2)_ = 1.72, *p* = 0.424
Dindo–Clavien					χ^2^_(2)_ = 1.03, *p* = 0.596	χ^2^_(2)_ = 3.34, *p* = 0.188
0 (zero)	136 (48.0)	47 (40.5)	34 (45.3)	55 (60.4)		
Minor (1–2)	83 (29.0)	45 (38.8)	23 (30.7)	15 (16.5)		
Major (≥3a)	63 (22.0)	24 (20.7)	18 (24.0)	21 (23.1)		
Hospital Readmission	57 (20.2)	24 (20.7)	13 (17.3)	20 (22.0)	χ^2^_(2)_ = 0.59, *p* = 0.745	χ^2^_(2)_ = 0.50, *p* = 0.780
Intraoperative Mortality	0 (0.0)	0 (0.0)	0 (0.0)	0 (0.0)	χ^2^_(2)_ = 0.00, *p* = 1.000	χ^2^_(2)_ = 1.32, *p* = 0.518
90 days Mortality	12 (4.3)	3 (2.6)	3 (4.0)	6 (6.6)	χ^2^_(2)_ = 1.98, *p* = 0.371	χ^2^_(2)_ = 3.13, *p* = 0.209
90 days Mortality in case of POPF type C	5 (1.8)	1 (0.9)	2 (2.7)	2 (2.2)	χ^2^_(2)_ = 0.77, *p* = 0.682	χ^2^_(2)_ = 3.13, *p* = 0.209

Values in parenthesis are percentages unless indicated otherwise. Likelihood ratio chi-square tests: ^†^ binomial regression and ^‡^ binomial survey-weighted regression, respectively. tsPJN: Neuhaus-style, telescope pancreatojejunostomy; PG: pancreatogastrostomy; dtmPJB: modified Blumgart-style, duct-to-mucosa pancreatojejunostomy; POPF: postoperative pancreatic fistula; ERCP: endoscopic retrograde cholangiopancreatography; DGE: delayed gastric emptying; PPH: post-pancreatectomy hemorrhage.

## Data Availability

The datasets used and/or analyzed during the current study are available from the corresponding author upon reasonable request.

## Supplementary Materials

The following supporting information can be downloaded at: https://www.mdpi.com/article/10.3390/jcm12093318/s1, Table S1: Histologic Diagnosis; Figure S1: Distribution of the performed pancreatic anastomosis techniques.
