# Supplementary material for: Single Center, Propensity Score Matching Analysis of Different Reconstruction Techniques following Pancreatoduodenectomy
Source: J Clin Med. 2023 May 6;12(9):3318. doi: 10.3390/jcm12093318 (PMC10179219; doi:10.3390/jcm12093318)
Supplement: Supplementary file 1 [file jcm-12-03318-s001.zip › Description Figure S1.pdf]

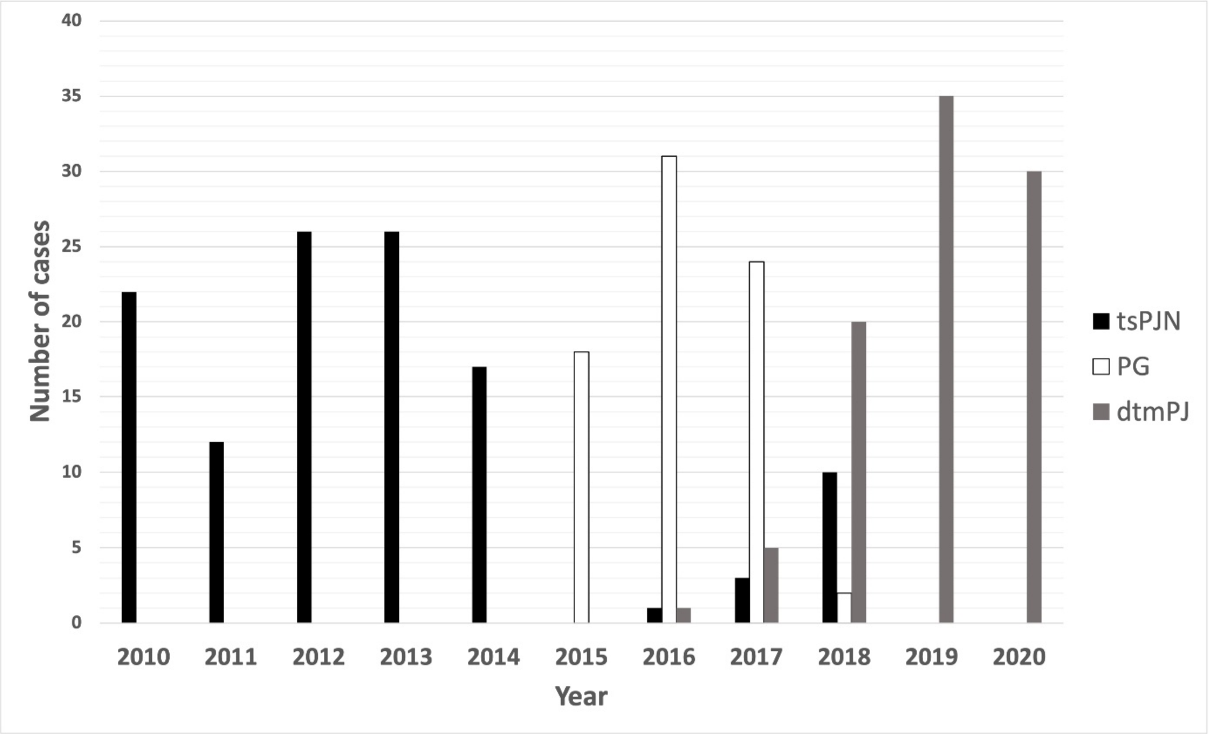

Figure S1. Distribution of the performed pancreatic anastomosis techniques at the Department for Visceral, Transplantation and Thoracic Surgery of the Medical University of Innsbruck from 2010 to 2020.
