# Supplementary material for: Single Center, Propensity Score Matching Analysis of Different Reconstruction Techniques following Pancreatoduodenectomy
Source: J Clin Med. 2023 May 6;12(9):3318. doi: 10.3390/jcm12093318 (PMC10179219; doi:10.3390/jcm12093318)
Supplement: Supplementary file 1 [file jcm-12-03318-s001.zip › Table S1.pdf]

Table S1. Histologic Diagnosis.

	All (n=282)	tsPJN (n=116)	PG (n=75)	dtmPJB (n=91)
ASC of the common bile duct	1 (0.4)	0 (0.0)	0 (0.0)	1 (1.1)
ASC of the pancreas	2 (0.7)	0 (0.0)	1 (1.3)	1 (1.1)
Autoimmune pancreatitis	2 (0.7)	1 (0.9)	0 (0.0)	1 (1.1)
dCCC	26 (9.2)	7 (6.0)	6 (8.0)	13 (14.1)
Chronic pancreatitis	23 (8.2)	14 (12.1)	3 (4.0)	6 (6.6)
Duodenal adenocarcinoma	9 (3.2)	4 (3.4)	1 (1.3)	4 (4.4)
GIST	1 (0.4)	1 (0.9)	0 (0.0)	0 (0.0)
Granulomatous inflammation	4 (1.4)	2 (1.7)	2 (2.7)	0 (0.0)
Haemangioma	1 (0.4)	1 (0.9)	0 (0.0)	0 (0.0)
IPMN	27 (9.6)	7 (6.0)	12 (16.0)	8 (8.8)
ITPN	1 (0.4)	0 (0.0)	1 (1.3)	0 (0.0)
Metastasis	4 (1.4)	0 (0.0)	2 (2.7)	2 (2.2)
MINEN	1 (0.4)	0 (0.0)	0 (0.0)	1 (1.1)
NEN	9 (3.2)	4 (3.4)	1 (1.3)	4 (4.4)
Pancreas divisum	1 (0.4)	0 (0.0)	1 (1.3)	0 (0.0)
PanIN	2 (0.7)	1 (0.9)	1 (1.3)	0 (0.0)
Papillary carcinoma	28 (9.9)	16 (13.8)	10 (13.3)	2 (2.2)
Papillary duodenal adenoma	3 (1.1)	0 (0.0)	0 (0.0)	3 (3.3)
PDAC	129 (45.7)	52 (44.8)	33 (44.0)	44 (48.4)
SCN	6 (2.1)	4 (3.4)	1 (1.3)	1 (1.1)
SPC	1 (0.4)	1 (0.9)	0 (0.0)	0 (0.0)
SPN	1 (0.4)	1 (0.9)	0 (0.0)	0 (0.0)

Values in parenthesis are percentages unless; ASC: adenosquamous carcinoma; dCCC: distal cholangiocellular carcinoma; GIST: gastrointestinal stroma tumor of the stomach; IPMN: intraductal papillary mucinous neoplasm; ITPN: intraductal tubulo-papillary neoplasm; MINEN: mixed neuroendocrine non-neuroendocrine neoplasm; NEN: neuroendocrine neoplasm; PanIN: pancreatic intraepithelial neoplasm; PDAC: pancreatic ductal adenocarcinoma ; SCN: serous cystic neoplasm; SPC: simple pan reatic cyst; SPN: solid pseudopapillary neoplasm.
